# Hearing Recovery Induced by DNA Demethylation in a Chemically Deafened Adult Mouse Model

**DOI:** 10.3389/fncel.2022.792089

**Published:** 2022-02-17

**Authors:** Xin Deng, Zhengqing Hu

**Affiliations:** ^1^Department of Otolaryngology-Head and Neck Surgery (HNS), Wayne State University School of Medicine, Detroit, MI, United States; ^2^John D. Dingell VA Medical Center, Detroit, MI, United States

**Keywords:** espin, otoacoustic emission, prestin, ribbon synapse, hair cell, auditory brainstem response (ABR), DNA demethylation

## Abstract

Functional hair cell regeneration in the adult mammalian inner ear remains challenging. This study aimed to study the function of new hair cells induced by a DNA demethylating agent 5-azacytidine. Adult mice were deafened chemically, followed by injection of 5-azacytidine or vehicle into the inner ear. Functionality of regenerated hair cells was evaluated by expression of hair cell proteins, auditory brainstem response (ABR), and distortion-product otoacoustic emission (DPOAE) tests for 6 weeks. In the vehicle-treated group, no cells expressed the hair cell-specific protein myosin VIIa in the cochlea, whereas numerous myosin VIIa-expressing cells were found in the 5-azacytidine-treated cochlea, suggesting the regeneration of auditory hair cells. Moreover, regenerated hair cells were co-labeled with functional proteins espin and prestin. Expression of ribbon synapse proteins suggested synapse formation between new hair cells and neurons. In hearing tests, progressive improvements in ABR [5–30 dB sound pressure level (SPL)] and DPOAE (5–20 dB) thresholds were observed in 5-azacytidine-treated mice. In vehicle-treated mice, there were <5 dB threshold changes in hearing tests. This study demonstrated the ability of 5-azacytidine to promote the functional regeneration of auditory hair cells in a mature mouse model *via* DNA demethylation, which may provide insights into hearing regeneration using an epigenetic approach.

## Introduction

According to the WHO, more than 1.5 billion people experience various extents of hearing disorders in the world (World Health Organization, 2021), which largely attribute to auditory hair cell loss and dysfunction. The mammalian cochlear hair cell epithelium has two types of hair cells: inner and outer hair cells. Inner hair cells transfer auditory information to spiral ganglion neurons *via* hair cell ribbon synapses. The neurotransmission between inner hair cells and spiral ganglion neurons involves C-terminal-binding proteins (CtBPs) family of transcriptional corepressor C-terminal-binding protein 2 (CtBP2) expressed at the presynaptic ribbon structure (Nouvian et al., 2006; Matthews and Fuchs, 2010; Uthaiah and Hudspeth, 2010; Liberman et al., 2011) and glutamate receptor 2 (GluR2) as one of the postsynaptic α-amino-3-hydroxy-5-methyl-4-isoxazolepropionic acid (AMPA)-type glutamate receptors (Nouvian et al., 2006; Uthaiah and Hudspeth, 2010; Liberman et al., 2011). Outer hair cells are able to detect weak sound vibrations that are attributed to cochlear amplification, likely *via* the endowed electromotility (Hudspeth, 1989, 2008). Prestin is a motor protein that is robustly expressed in outer hair cells and acts as an amplifier of sound stimulations (Zheng et al., 2000a; Dallos, 2008). In the complex mammalian auditory system, the functionality of hair cells is carried out by the stereocilia of hair bundles, which consist of a variety of proteins, including actin cross-linking proteins, such as espin (Hudspeth, 1989, 2008; Bartles, 2000), as well as the abovementioned functional proteins to detect sound stimulation and transfer signals to spiral ganglion neurons. However, mammalian auditory system is vulnerable to a variety of insults, which causes irreversible degeneration of auditory hair cell functional proteins and loss of hair cells, leading to permanent sensorineural hearing loss (Hawkins et al., 1976; Bohne et al., 1987). Therefore, a successful hair cell regeneration includes not only the reconstitution of structure and morphology, but also the regeneration of hair cell functional proteins and synaptic connections.

In the past decades, research on hair cell regeneration has gained significant progress. Regenerated hair cells are observed in the *in vitro* system (White et al., 2006; Oshima et al., 2010; Chai et al., 2012; Chen et al., 2012; György et al., 2017; Koehler et al., 2017) and neonatal *in vivo* models (White et al., 2006; Chai et al., 2012; György et al., 2017; Isgrig et al., 2017). Gene therapy is shown to repair the function of hair cells in the neonatal mouse (Isgrig et al., 2017; Nist-Lund et al., 2019) and rescue the deafness phenotype in the postnatal mouse (György et al., 2017). In an *in vitro* study, the gerbil organ of Corti was cultured in the presence of a Notch signaling inhibitor. Supporting cell stereocilia formation and native hair cell supernumerary stereocilia were identified, which showed mechanotransduction currents following fluid jet stimulation (Li et al., 2018). In addition, Notch inhibition induced new hair cells to express prestin, a major characteristic protein of outer hair cells that mediates somatic mobility (Santos-Sacchi and Dilger, 1988; Zheng et al., 2000b; Liberman et al., 2002). *In vivo*, hair bundle-like structures and possible nerve connection adjacent to newly generated hair cell-like cells were induced by ectopic expression of Atonal homolog 1 (*Atoh1*), POU class 4 transcription factor 3 (*Pou4f3*), and GATA-binding protein 3 (*Gata3*) in the presence of *P27*^*Kip*1^ inhibition. In the other *in vivo* study, new hair cell-like cells expressed prestin, CtBP2, and vesicular glutamate transporter 3 (VGluT3) and showed FM1-43 intake following *Atoh1*, *Pou4f3*, and growth factor independence-1 (*Gfi1*) overexpression (Chen et al., 2021). However, the overall hearing threshold was worsened, indicating the hearing function recovery is still challenging (Chen et al., 2021). These previous reports demonstrate promising, but limited progress on hair cell function regeneration, suggesting that regeneration of functional hair cells in adult mammals remains a major obstacle.

In this study, we attempted to investigate the functionality of newly generated hair cells induced by a previously established epigenetic approach in a deafened adult mouse model (Deng et al., 2019; Deng and Hu, 2020). The advantage of an epigenetic approach is that this approach does not change the DNA sequence (Jones and Takai, 2001; Layman and Zuo, 2015), which may be critical for future clinical application. In our previous report, a DNA demethylating agent 5-azacytidine (5-aza) was able to stimulate mouse utricle sensory epithelia-derived progenitor cells (MUCs) to differentiate into hair cell-like cells expressing *Atoh1*, myosin VIIa, myosin VI, espin, and Pou4f3 *in vitro* (Zhou and Hu, 2016). FM1-43 permeation assay revealed that the 5-aza-treated MUCs may possess functional mechanosensory transduction channels (Zhou and Hu, 2016). Later, 5-aza was injected into the inner ear of the deafened adult mouse to investigate whether 5-aza could promote hair cell regeneration *in vivo* (Deng et al., 2019). Two weeks following 5-aza treatment, regenerated hair cells were identified in the cochlear sections and basilar membrane surface preparations, which were confirmed by myosin VIIa immunostaining and fluorescent F-actin double-labeling (Deng et al., 2019). The regenerated hair cells were observed in all the three cochlear turns and were able to survive for at least 6 weeks (Deng et al., 2019). Moreover, the inner ear DNA methyltransferase 1 (Dnmt1) messenger RNA (mRNA) expression level of the 5-aza treatment group was significantly lower than that of the vehicle treatment group, suggesting that DNA demethylation and Dnmt1 inhibition may play a role in 5-aza-induced new hair cell generation (Deng et al., 2019). In order to determine the cell sources for the new hair cells, a transgenic mouse strain that the expression of enhanced green fluorescent protein (EGFP) is under control of the endogenous SRY-Box transcription factor 2 (Sox2) regulatory element (the Sox2-EGFP transgenic mouse) was applied. The results indicate that the new hair cells may be converted from Sox2-positive supporting cells 5–7 days post-5-aza injection (Deng and Hu, 2020). Further, CtBP2 expression observed in new hair cells suggests that these 5-aza-induced hair cells may have the potential to form hair cell ribbon synapses (Deng et al., 2019). These data motivated us to further the study to investigate the function of new hair cells generated by 5-aza treatment *in vivo*.

## Materials and Methods

### Animals

Young adult (4–6 weeks old, either sex) wildtype Swiss Weber mice were included in this study. The care and use of animals have been approved by the Wayne State University Institutional Animal Care and Use Committee. A total of 48 animals were included in the surgery, 24 animals for the 5-aza and 24 animals for the vehicle groups. Six animals were assigned to the 1, 2, 4, and 6 weeks postsurgery groups for the 5-aza and vehicle groups, respectively. Additionally, six untreated normal hearing mice were included in the synapse study.

### Hearing Test

Auditory brainstem response (ABR) and distortion-product otoacoustic emission (DPOAE) tests are two major methods used to determine the function of the auditory system and outer hair cells, respectively (Tadros et al., 2007a,b). The five waveforms of ABR test provide information about the auditory nerve and different cell types in the cochlear nucleus and superior olivary complex (Tadros et al., 2007a,b). DPOAE measures the amplification of cochlear vibration generated by outer hair cells (Tadros et al., 2007a,b). Thus, a combination of ABR and DPOAE provides a functional status of the hair cells.

Baseline pure-tone ABR and DPOAE were used to evaluate the auditory and outer hair cell function using our published methods (Deng et al., 2019; Hu et al., 2021). ABR and DPOAE were measured using RP2.1 and RZ6 systems [Tucker-Davis Technology (TDT), Alachua, FL, United States]. The TDT System 3 software was utilized for signal generation and response collection. At 8, 16, 24, and 32 kHz, the ABR stimulation ranged from 10 to 90 dB sound pressure level (SPL) in 5 dB steps. The threshold was determined as the lowest stimulation dB SPL level that generated wave-I amplitude larger than 0.2 μV (microvolt). At 16 and 24 kHz, the configuration of DPOAE was F2/F1 at 1.2, L1 = L2 + 10 dB. L1 ranged from 10 to 90 dB SPL in 5 dB SPL steps. DPOAE threshold was determined as the lowest level with DPOAE responses (dp) of at least 10 dB above the noise floor.

Auditory brainstem response and DPOAE tests were performed prior to deafening to record the baseline, 3-day postdeafening to confirm deafness, and at each week following surgery for up to 6 weeks to determine hearing ability recovery. Since all the follow-up animals received weekly hearing tests after the surgery, 24, 18, 12, and 6 animals received hearing tests at 1, 2, 4, and 6 weeks following 5-aza treatment, respectively. Similarly, 24, 18, 12, and 6 animals received hearing tests at 1, 2, 4, and 6 weeks after surgery in the vehicle group, respectively.

### Deafening

Baseline pure-tone ABR and DPOAE were performed on each mouse to assure normal hearing. The deafening was performed using our published methods (Deng et al., 2019). Briefly, kanamycin (1 g/kg body weight, sc) was injected, followed by furosemide treatment (300 mg/kg body weight, ip) after 25–30 min (Deng et al., 2019). Deafness was confirmed 3-day after kanamycin and furosemide injection. ABR threshold shift larger than 40 dB SPL was set as criteria of successful deafening.

### Inner Ear Surgery

At 3 days postdeafening, 4 mM 5-aza was injected into deafened mouse inner ears after hearing tests using our published methods (Deng et al., 2019). The control group was injected with saline, which was the vehicle. Under deep anesthesia, the left temporal bone was exposed and opened to enable visuality of the basal cochlear turn and round window. A volume of 0.2 μl of 5-aza or saline was injected *via* a microsyringe (Hamilton, Reno, NV, United States), which was connected to a small catheter inserting into the round window. Mice were followed up for 6 weeks, with ABR and DPOAE tests weekly.

### Immunofluorescence

At the end of the experiment, mice were euthanized and cochlear samples were harvested and fixed by 4% paraformaldehyde (Sigma-Aldrich, Burlington, MA, United States) using our published methods (Deng et al., 2019). Cochleae were decalcified by 0.1 M ethylenediaminetetraacetic acid (EDTA) (Sigma-Aldrich) for 1 week. After decalcification, cochleae were cryosectioned at 10 μm thickness. For immunostaining, cochlear samples were treated with phosphate-buffered saline (PBS) containing 5% normal donkey serum (Jackson ImmunoResearch, West Grove, PA, United States) and 0.2% Triton X-100 (Sigma-Aldrich) for 30 min. Cochlear sections were incubated in primary antibodies at 4°C overnight, followed by secondary antibody incubation at room temperature for 2 h. The primary antibodies included: anti-myosin VIIa [1:100; Developmental Studies Hybridoma Bank (DSHB), Iowa City, IA, United States and Proteus Biosciences, Ramona, CA, United States], anti-espin (1:50; gift of Dr. James Bartels), anti-prestin (1:50, Sigma-Aldrich), anti-CtBP2 (1:200, BD Biosciences, San Jose, CA, United States), and anti-GluR2 (1:200, Millipore, Burlington, MA, United States). Secondary antibodies included Dylight- 488-, Cy3-, and Dylight-647-conjugated antibodies (1:500; Jackson ImmunoResearch, West Grove, PA, United States). 4,6-diamidino-2-phenylindole (DAPI) (Invitrogen, Waltham, MA, United States) was used to label all the nuclei. Leica confocal microscopy was used to capture images.

### Data Analysis

The expressions of CtBP2 and GluR2 were quantified by counting the number of puncta per inner hair cell within the inner hair cell area. Data were sampled from the normal hearing mice and 5-aza-treated mice. Because no or very few inner hair cells were observed in the vehicle group, vehicle-treated mice were not included in the synapse study analysis. Statistical analysis was performed by Tukey’s honestly significant difference (HSD) test. *p* < 0.05 was considered as statistically significant in this study.

To evaluate the hearing function of the 5-aza- or vehicle-treated mice, ABR and DPOAE threshold shifts were calculated by the thresholds measured postsurgery, subtracting those at 3 days postdeafening, which was just before the surgery. Data were shown by mean ± SEM. Lower thresholds indicated better hearing function and the negative threshold shifts suggested a decrease in the thresholds and improvement of hearing. Two-way ANOVA followed by Tukey’s test was used to define the threshold shift difference between the vehicle- and 5-aza-treated mice among different follow-up points. *p* < 0.05 was considered as statistically significant in this study.

## Results

### Hair Cell Functional Proteins Were Expressed Following 5-Aza Treatment

**FIGURE 1:**
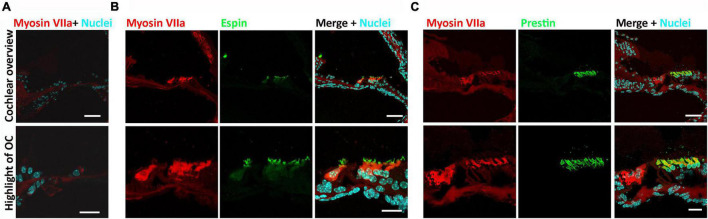
Expression of espin and prestin was observed in the 5-azacytidine (5-aza)-treated mouse inner ear. **(A)** No hair cell was observed in the cochlear sections of the vehicle-treated mice. **(B)** Expression of espin was observed in the hair bundles of myosin VIIa-expressing regenerated hair cells in the 5-aza-treated mice. **(C)** Prestin expressed in the myosin VIIa-expressing outer hair cells in the cochlear sections of the 5-aza group mouse. All the figures were from the apical cochlear turn. OC, organ of Corti. Scale bar: 50 μm in cochlear overview and 20 μm in OC highlight.

Administration of kanamycin and furosemide (see section “Materials and Methods”) was consistently shown to deafen young adult mice (4–6 weeks old) (Deng et al., 2019; Deng and Hu, 2020). Pure-tone ABR and DPOAE were measured prior to and 3 days after deafening to confirm deafness, as shown in our previous reports (Deng et al., 2019; Deng and Hu, 2020). Two weeks after 5-aza or vehicle injection, cochlear section immunostaining was performed to observe the expression of hair cell functional proteins. Previous reports utilized expressions of espin in hair cell bundle stereocilia (Bartles, 2000) and prestin in the outer hair cells (Zheng et al., 2000a; Dallos, 2008) to evaluate the functionality of hair cells. In this study, no myosin VIIa-expressing cell was found in vehicle-treated mice (Figure 1A), suggesting no hair cell was regenerated in the vehicle group, which is consistent with our previous report (Deng et al., 2019). In contrast, myosin VIIa-expressing hair cells were observed in the 5-aza-treated mice (Figures 1B,C), confirming hair cells regeneration induced by 5-aza. All the myosin VIIa-expressing hair cells were colabeled with espin (Figure 1B), indicating the expression of hair bundle proteins in these new hair cells. Expression of prestin was also observed in myosin VIIa-expressing hair cells and all the myosin VIIa-positive cells in the outer hair cell region were prestin positive (Figure 1C), suggesting the recovery of outer hair cell functional proteins.

### Hair Cell Ribbon Synapse Proteins Were Expressed Following 5-Aza Treatment

**FIGURE 2:**
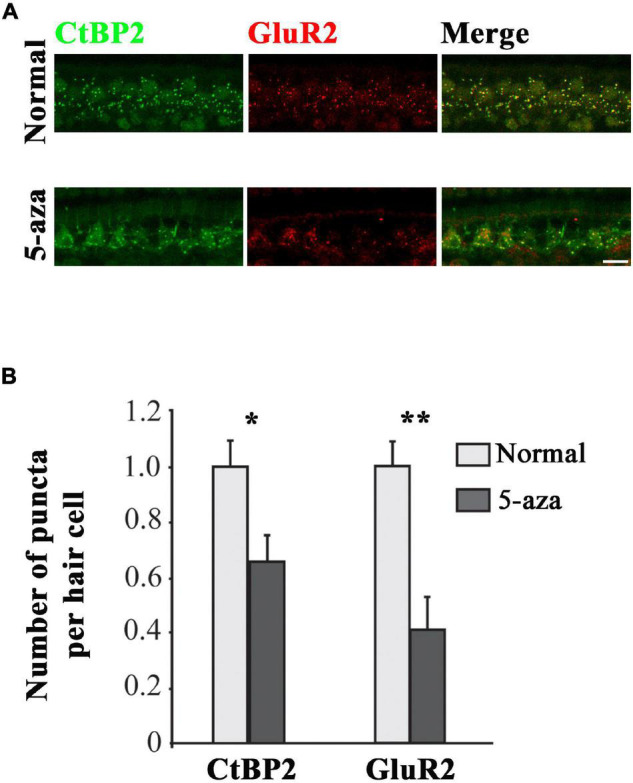
Expression of C-terminal-binding protein 2 (CtBP2) and GluR2 was observed in the 5-aza-treated mice. **(A)** Expression of CtBP2 and GluR2 in the surface preparation of the basilar membrane of the normal hearing and 5-aza-treated mice. Scale bar: 50 μm. **(B)** Quantification of CtBP2 and GluR2 expression. The number of CtBP2 and GluR2 puncta per inner hair cell was normalized to 1 for the normal hearing mouse. Data are means ± SEM. Statistical analysis was performed by Tukey’s honestly significant difference (HSD) test. * indicates *p* < 0.05; **indicates *p* < 0.01.

C-terminal-binding protein 2 (CtBP2) is the ribbon synapse vesicle protein of hair cell afferent synapses (Uthaiah and Hudspeth, 2010) and GluR2 is the postsynaptic AMPA-type glutamate receptors (Liberman et al., 2011). In this study, anti-CtBP2 and anti-GluR2 antibodies were applied to the basilar membrane to immunolabel these synaptic proteins to evaluate their expression in inner hair cell afferent ribbon synapses at 2 weeks after 5-aza treatment (Figure 2). CtBP2 and GluR2 puncta were observed in all the three cochlear turns in the 5-aza-treated mice. Figure 2A shows CtBP2 and GluR2 immunofluorescence labeling in the middle turn of normal hearing and the 5-aza treated mice. CtBP2 and adjacent GluR2 expression were observed in the inner hair cell region of both the 5-aza and normal hearing groups (Figure 2A). Merged figures revealed that some of CtBP2 and GluR2 signals were colocalized (Figure 2A). The quantitative study compared the expression of CtBP2 and GluR2 in the normal hearing and 5-aza groups (Figure 2B). In the quantification study, the average number of CtBP2 and GluR2 puncta per inner hair cell in the normal hearing group was normalized to 1.0 (Figure 2B). The average number of CtBP2 puncta per inner hair cell in the 5-aza group was 0.66 ± 0.09 (Figure 2B). Statistical analysis showed significant difference in the number of CtBP2 puncta between the normal hearing and 5-aza groups (*p* = 0.022; Figure 2B). After normalizing the GluR2 puncta in normal hearing mice, the number of GluR2 puncta per inner hair cell of the 5-aza group was 0.41 ± 0.12 (Figure 2B). Statistical analysis shows significant difference in GluR2 puncta between the normal and 5-aza groups (*p* = 0.001; Figure 2B). These data indicate that functional synaptic proteins have been regenerated in the 5-aza group. However, these 5-aza-induced synaptic protein expression has not been fully recovered to the normal hearing level.

### Auditory Brainstem Response Thresholds Were Improved by 5-Aza Treatment

**FIGURE 3:**
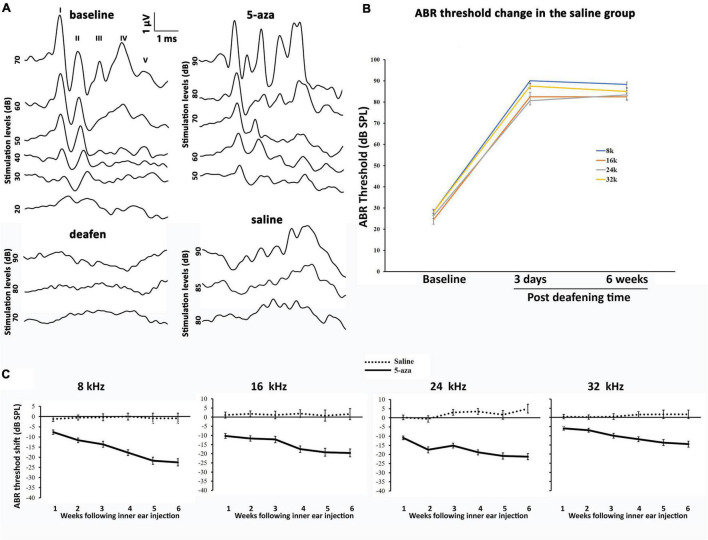
Auditory brainstem response (ABR) thresholds in the 5-aza- and vehicle-treated mice. **(A)** Representative ABR waveforms of four groups of mice. ABR threshold was determined by the lowest stimulation dB sound pressure level (SPL) that generates the amplitude of wave I greater than 0.2 μV. The baseline threshold was about 30 dB in this figure. Three days after deafening, deafened mice showed no ABR response up to 90 dB SPL stimulation. Two weeks after 5-aza treatment, ABR trace showed a threshold of 50 dB SPL. In contrast, the vehicle-treated mouse showed messy traces without typical ABR waveform responses. **(B)** ABR threshold shifts were over 40 dB SPL 3 days after deafening in the saline group. The threshold shifts remained 6 weeks following deafening. **(C)** ABR threshold shifts trend for up to 6 weeks after surgery in the 5-aza and vehicle groups at 8, 16, 24, and 32 kHz. Negative values in the 5-aza group revealed hearing improvement. ABR threshold shifts in the vehicle group were close to zero, indicating that vehicle injection did not significantly change ABR threshold. Data are means ± SEM.

Auditory brainstem response was applied to acquire functional information on the cochlea and auditory pathway (Squires et al., 1978; Parham et al., 1999). Baselines pure-tone ABRs were measured to ascertain that all the animals included in this study showed normal hearing ability. The baseline pure-tone ABR thresholds in both the 5-aza and vehicle mice were approximately 10–40 dB SPL at 8, 16, 24, and 32 kHz (Figures 3A,B). Three days after kanamycin/furosemide treatment, ABR thresholds of the same mice were >70 dB SPL at 8, 16, 24, and 32 kHz (Figure 3A), indicating that mice were chemically deafened. Vehicle-treated animals did not show significant ABR threshold changes up to 6 weeks after vehicle injection into the cochleae (Figures 3A,B).

In contrast, mice treated with 4 mM 5-aza showed improved thresholds after surgery (Figure 3C), suggesting that 5-aza treatment may lead to hearing function improvement. To quantify hearing improvements, ABR thresholds were measured each week for up to 6 weeks after the 5-aza or vehicle treatment. The threshold shifts were calculated as threshold measured each week for up to 6 weeks subtracting the threshold prior to the 5-aza or vehicle treatment (see section “Materials and Methods”). Table 1 shows ABR threshold shifts at different frequencies in 5-aza and vehicle mice from 1- to 6-week following treatment. In contrast to the vehicle-treated mice with overall ABR threshold changes around −2 to 5 dB SPL, the 5-aza-treated mice exhibited progressive ABR threshold improvement for up to 30 dB SPL from 1 to 6-week following treatment in all the frequencies measured (Table 1 and Figure 3C).

**TABLE 1:** Auditory brainstem response (ABR) threshold shifts after 5-aza/vehicle treatment.

Frequency (kHz)	Group	1-week	2-week	3-week	4-week	5-week	6-week
8	5-aza	−8.125 ± 1.137	−12.222 ± 1.312	−13.75 ± 1.607	−16.25 ± 1.607	−24.167 ± 2.273	−30.833 ± 2.273
	Vehicle	−1.042 ± 1.137	−0.556 ± 1.312	−0.417 ± 1.607	−0.001 ± 1.607	−0.833 ± 2.273	−0.833 ± 2.273
16	5-aza	−10.625 ± 1.491	−11.944 ± 1.722	−11.25 ± 2.109	−17.083 ± 2.109	−20.833 ± 2.982	−24.167 ± 2.982
	Vehicle	1.042 ± 1.491	1.389 ± 1.722	0.417 ± 2.109	2.083 ± 2.109	0.833 ± 2.982	1.667 ± 2.982
24	5-aza	−11.875 ± 1.076	−16.389 ± 1.242	−17.083 ± 1.522	−20.417 ± 1.522	−22.5 ± 2.152	−22.5 ± 2.152
	Vehicle	0.417 ± 1.076	3.056 ± 1.242	1.25 ± 1.522	4.583 ± 1.522	1.667 ± 2.152	1.667 ± 2.152
32	5-aza	−5.833 ± 1.021	−8.611 ± 1.178	−11.25 ± 1.443	−12.5 ± 1.443	−17.5 ± 2.041	−22.5 ± 2.041
	Vehicle	0.208 ± 1.201	0.833 ± 1.178	0.001 ± 1.443	1.667 ± 1.443	1.667 ± 2.041	1.667 ± 2.041

*Threshold shift (dB SPL) was calculated as ABR threshold measured after the treatment minus the threshold prior to the treatment (3 days after deafening). Data are means ± SEM.*

Statistical analysis revealed that ABR threshold improvements in 5-aza and vehicle mice were significantly different at all the follow-up points at all the frequencies using two-way ANOVA and *post-hoc* tests. The two factors are treatment type (5-aza and vehicle) and follow-up times (1–6 weeks). At 8 kHz, the overall effects were *F*_(5, 144)_ = 10.82 and *p* < 0.0001, whereas the effects of treatment type and follow-up time were *F*_(1, 144)_ = 279.2/*p* < 0.0001 and *F*_(5, 144)_ = 10.31/*p* < 0.0001, respectively. In *post-hoc* tests of comparing the 5-aza and vehicle groups, *p* < 0.0001 was observed from 1 to 6 weeks posttreatment. At 16 kHz, two-way ANOVA showed that the overall, treatment type, and follow-up time effects were significant, *F*_(5, 144)_ = 3.003/*p* = 0.0131, *F*_(1, 144)_ = 189.5/*p* < 0.0001, and *F*_(5, 144)_ = 2.401/*p* = 0.0399, respectively. In *post-hoc* tests, the 5-aza vs. vehicle groups comparison showed *p* < 0.0001 for the 1, 2, 4, 5, and 6 weeks measurements, whereas *p* = 0.0004 for the 3 weeks measurements. At 24 kHz, two-way ANOVA analysis revealed that the effects of overall, treatment type, and follow-up times were statistically significant: *F*_(5, 144)_ = 9.840/*p* < 0.0001, *F*_(1, 144)_ = 534.8/*p* < 0.0001, and *F*_(5, 144)_ = 3.107/*p* = 0.0108, respectively. In *post-hoc* comparison of the 5-aza and vehicle groups 1–6 weeks after the treatment, *p*-values were all < 0.0001. At 32 kHz, two-way ANOVA analysis revealed significant difference (*p* < 0.0001) in the overall, treatment type, and follow-up time effects: *F*_(5, 144)_ = 8.756, *F*_(1, 144)_ = 237.6, and *F*_(5, 144)_ = 5.915, respectively. In *post-hoc* comparison of the 5-aza and vehicle groups 1–6 weeks following treatment, *p* = 0.0003 for the 1-week follow-up and *p* < 0.0001 for the 2–6 weeks follow-ups.

To compare the 5-aza group threshold improvement among follow-up times, Table 2 shows the statistical analysis of ABR threshold shift 1–6 weeks post-5-aza treatment. In all frequencies, ABR threshold improvements of the 5- and 6-week groups were significantly greater than the 1- and 2-week groups (Table 2). For the vehicle group, there was no significant difference in all the comparisons at all the frequencies.

**TABLE 2:** Statistical analysis of weekly auditory brainstem response (ABR) threshold shifts of the 5-aza group.

	8 kHz	16 kHz	24 kHz	32 kHz
6-week	vs. 1-week (***); vs. 2-week (***); vs. 3-week (***); vs. 4-week (***); vs. 5-week (0.301)	vs. 1-week (***); vs. 2-week (**); vs. 3-week (**); vs. 4-week (0.378); vs. 5-week (0.969)	vs. 1-week (***); vs. 2-week (**); vs. 3-week (**); vs. 4-week (0.969); vs. 5-week (1.000)	vs. 1-week (***); vs. 2-week (***); vs. 3-week (***); vs. 4-week (***); vs. 5-week (0.510)
5-week	vs. 1-week (***); vs. 2-week (***); vs. 3-week (**); vs. 4-week (0.051)	vs. 1-week (**); vs. 2-week (0.102); vs. 3-week (0.091); vs. 4-week (0.909)	vs. 1-week (***); vs. 2-week (0.136); vs. 3-week (0.311); vs. 4-week (0.969)	vs. 1-week (***); vs. 2-week (**); vs. 3-week (0.124); vs. 4-week (0.342)
4-week	vs. 1-week (***); vs. 2-week (0.377); vs. 3-week (0.882)	vs. 1-week (0.124); vs. 2-week (0.401); vs. 3-week (0.368)	vs. 1-week (***); vs. 2-week (0.314); vs. 3-week (0.632)	vs. 1-week (**); vs. 2-week (0.294); vs. 3-week (0.717)
3-week	vs. 1-week (*); vs. 2-week (0.977)	vs. 1-week (1.000); vs. 2-week (1.000)	vs. 1-week (0.058); vs. 2-week (0.999)	vs. 1-week (*); vs. 2-week (0.249)
2-week	vs. 1-week (0.17)	vs. 1-week (0.992)	vs. 1-week (0.066)	vs. 1-week (0.478)

Pairwise comparison was performed by the Tukey test. P-values were stated in parentheses. *, **, and *** indicate p < 0.05, p < 0.01, and p < 0.001, respectively.

Taken together, statistical analysis revealed that ABR threshold improvements in the 5-aza and vehicle groups were significantly different at all follow-up timepoints and all frequencies (*p* < 0.001 in all the comparisons), suggesting 5-aza-induced hearing improvement for at least 6 weeks compared to the vehicle group.

### Distortion-Product Otoacoustic Emission Thresholds Were Improved by 5-Aza Treatment

**FIGURE 4:**
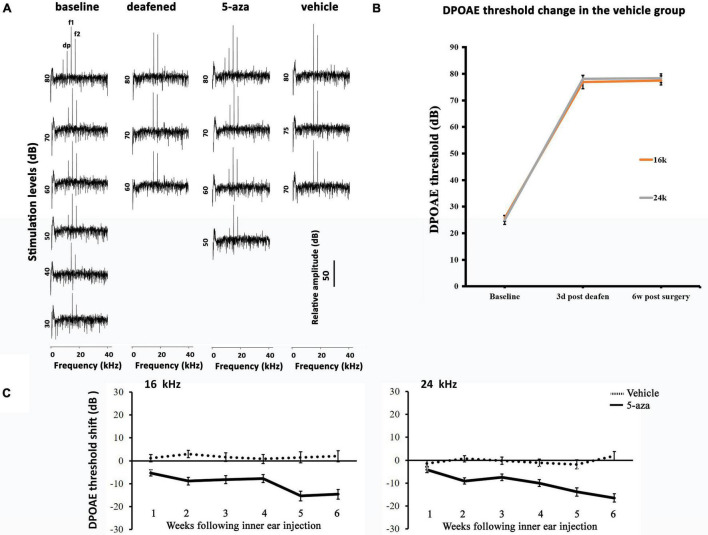
Distortion-product otoacoustic emission (DPOAE) thresholds in the 5-aza- and vehicle-treated mice. **(A)** Representative DPOAE traces of 4 groups of mice. DPOAE threshold was determined by the lowest stimulation dB level that generates dp of at least 10 dB above the noise floor. The baseline threshold was 40 dB in this figure. Three days after deafening, DPOAE threshold was 80 dB. The 5-aza treated mouse showed 50 dB threshold 6 weeks after treatment, while the vehicle-treated mouse showed no DPOAE response 6 weeks after injection. **(B)** DPOAE threshold shifts were over 40 dB SPL 3 days after deafening in the saline group. The threshold shifts remained 6 weeks following deafening. **(C)** DPOAE threshold shifts trend for up to 6 weeks after surgery in the 5-aza and vehicle groups at 16 and 24 kHz. Negative values suggest hearing improvement. DPOAE threshold shifts in the vehicle group were close to zero, indicating that vehicle injection did not significantly change DPOAE threshold. Data are means ± SEM.

Distortion-product otoacoustic emissions at 16 and 24 kHz were applied to test the functionality of outer hair cells. In baseline tests, DPOAE thresholds were approximately 20–40 dB at 16 and 24 kHz (Figures 4A,B). Three days following kanamycin/furosemide treatment, DPOAE thresholds of the same mice went up to >50 dB (Figures 4A,B), indicating impairment of outer hair cells. After the treatment, the control group animals showed no significant DPOAE threshold changes up to 6 weeks after vehicle injection into the cochleae (Figure 4B). In contrast, mice treated with 4 mM 5-aza showed improved thresholds after the treatment (Figures 4A,C), suggesting that 5-aza treatment may lead to hearing function improvement. To evaluate outer hair cell function improvements, DPOAE thresholds of the 5-aza- and vehicle-treated mice were measured each week for up to 6 weeks following surgery and then compared against DPOAE thresholds right before the surgery (*n* = 6 mice per group; see section “Materials and Methods”). Similar to the analysis of ABR threshold shifts, DPOAE threshold improvements show a trend of 5–20 dB increase from 1 to 6 weeks after 5-aza injection (Figure 4C). In the vehicle-treated mice, limited DPOAE threshold shifts were observed after surgery in both frequencies (Figure 4B). Table 3 shows DPOAE threshold shifts in the 5-aza and vehicle groups.

**TABLE 3:** Distortion-product otoacoustic emission (DPOAE) threshold shifts 1–6 week*s* post*-*treatment.

Frequency (kHz)	Group	1-week	2-week	3-week	4-week	5-week	6-week
16	5-aza	−5.833 ± 1.346	−8.889 ± 1.554	−10 ± 1.904	−11.667 ± 1.904	−18.333 ± 2.692	−18.333 ± 2.692
	Vehicle	1.458 ± 1.346	1.667 ± 1.554	1.667 ± 1.904	0.417 ± 1.904	0.833 ± 2.692	1.667 ± 2.692
24	5-aza	−5.208 ± 1.267	−7.778 ± 1.463	−9.167 ± 1.792	−10.417 ± 1.792	−17.5 ± 2.534	−20 ± 2.534
	Vehicle	−0.833 ± 1.267	−0.278 ± 1.463	−0.833 ± 1.792	−0.833 ± 1.792	−4.167 ± 2.534	0.001 ± 2.534

*Threshold shift (dB) was calculated as DPOAE threshold measured weekly after treatment minus DPOAE threshold prior to the treatment (3 days after deafening). Data are means ± SEM.*

Two-way ANOVA analysis revealed significant difference at 16 kHz for the overall, treatment type (5-aza vs. vehicle), and follow-up period (1–6 weeks follow-up) effects: *F*_(5, 144)_ = 2.828/*p* = 0.0182, *F*_(1, 144)_ = 125.6/*p* < 0.0001, and *F*_(5, 144)_ = 3.250/*p* = 0.0082, respectively. These numbers became *F*_(5, 144)_ = 2.648/*p* = 0.0254, *F*_(1, 144)_ = 93.14/*p* < 0.0001, and *F*_(5, 144)_ = 6.293/*p* < 0.0001 for 24 kHz, respectively. In *post-hoc* comparison of the 5-aza and vehicle groups from 1 to 6 weeks follow-up at 16 kHz, *p*-values were 0.0011 and 0.0002 for 1- and 3-week posttreatment, whereas *p* < 0.0001 for 2-, 4-, 5-, and 6-week follow-ups. At 24 kHz, *p*-values were 0.0017, 0.0024, 0.0075, 0.0013, 0.0017, and <0.0001 for 1–6 weeks posttreatment in the comparison of the 5-aza and vehicle-treated groups, respectively.

To compare the 5-aza group threshold shift improvement among follow-up times, Table 4 shows the significant difference in *post-hoc* analysis in both frequencies. DPOAE threshold improvements of the 5- and 6-week groups showed greater threshold improvements than the first 2 weeks groups (Table 4). No significant difference was observed in all the comparisons within the vehicle group in either frequency.

**TABLE 4:** Statistical analysis of distortion-product otoacoustic emission (DPOAE) threshold shifts of the 5-aza group.

	16 kHz	24 kHz
6-week	vs. 1-week (***) vs. 2-week (*) vs. 3-week (0.116) vs. 4-week (0.330) vs. 5-week (1.000)	vs. 1-week (***) vs. 2-week (***) vs. 3-week (**) vs. 4-week (*) vs. 5-week (0.982)
5-week	vs. 1-week (***) vs. 2-week (*); vs. 3-week (0.116); vs. 4-week (0.330)	vs. 1-week (*); vs. 2-week (*); vs. 3-week (0.078); vs. 4-week (0.201)
4-week	vs. 1-week (0.123); vs. 2-week (0.869); vs. 3-week (0.990)	vs. 1-week (0.166); vs. 2-week (0.864); vs. 3-week (0.996)
3-week	vs. 1-week (0.474); vs. 2-week (0.998)	vs. 1-week (0.463); vs. 2-week (0.991)
2-week	vs. 1-week (0.673)	vs. 1-week (0.770)

Pairwise comparison was performed by the Tukey test. P-values were stated in parentheses. *, **, and *** indicate p < 0.05, p < 0.01, and p < 0.001, respectively.

## Discussion

In this study, DNA demethylating agent 5-aza was injected into chemically deafened mature mouse cochleae. Generation of new hair cells was observed in the 5-aza-treated mouse inner ear. The new hair cells expressed hair cell functional proteins, including espin and prestin. Expression of hair cell ribbon synaptic proteins CtBP2 and GluR2 was observed and analyzed. Functional evaluation by ABR and DPOAE assays revealed improved ABR and DPOAE thresholds in the 5-aza-treated mice, suggesting that these new hair cells may be functional. These data indicate that DNA demethylation facilitates the regeneration of functional hair cells in the young adult mouse.

The deafening method using the combination of kanamycin and furosemide in this study was shown to entirely damage outer hair cells at 3 days postdeafening (Figure 1A; Deng et al., 2019). In the 5-aza groups, outer hair cells were observed by the immunolabeling of myosin VIIa antibody 2–6 weeks after the treatment, indicating and confirming the regeneration of outer hair cells by the Dnmt1 inhibitor 5-aza (Figures 1B,C; Deng et al., 2019). The immunostaining of espin and prestin was both colabeled with myosin VIIa, indicating the functionality of hair bundles and outer hair cells by 5-aza treatment (Figures 1B,C). The irregular orientation observed from espin and prestin labeling is consistent with the previous results that the regenerated hair cell bundles were abnormal (Deng et al., 2019), suggesting recovered albeit compromised functionality of new hair cells.

Expression of CtBP2 and GluR2 puncta in myosin VIIa-expressing cells at 2 weeks following 5-aza treatment suggested the regeneration of hair cell ribbon synapse vesicles (Figure 2A). The numbers of CtBP2 and GluR2 puncta of the 5-aza-treated mice remained less than that of the normal hearing mice, suggesting that the synaptic connection of newly generated hair cells was not fully recovered. Notably, the 5-aza-treated mice showed the presence of hair cells in the inner hair cell region (Figure 2A). It has been found in our previous report that a few inner hair cells survived at 3 days after deafening and 5-aza was injected into the inner ear 3 days postdeafening (Deng et al., 2019). Therefore, in this and previous studies (Deng et al., 2019), hair cells observed at the inner hair cell region in the 5-aza-treated mice might be new hair cells or surviving/recovery of native inner hair cells, which deserves an independent study.

The hearing recovery was confirmed by ABR and DPOAE test. ABR assays showed the functional recovery of regenerated hair cells by 5-aza. Overall, the 5-aza-treated mice showed at least 5 dB SPL and no more than 30 dB SPL threshold improvements in pure-tone ABR measurements (Figure 3C). At all the studied frequencies ranging from 8 to 32 kHz, ABR thresholds exhibited a trend of increasing improvement from 1 to 6 weeks after 5-aza injection (Figure 3C), indicating that new hair cells with corresponding basilar membrane were able to respond to sound wave dispersion for their spatially separated frequencies selectivity. The greatest ABR threshold improvement was at 8 kHz and the smallest ABR threshold improvement was at 32 kHz (Figure 3C). These data were consistent with the previous report studying the outer hair cell regeneration in different cochlear turns (Deng et al., 2019), in which the majority of regenerated outer hair cells were in the apex and middle turns (Deng et al., 2019). Notably, in all investigated frequencies, significantly greater threshold improvements were usually observed 4–5 weeks post-5-aza treatment, suggesting that the functionality of regenerated hair cells became better over time, while during 1–3 weeks after 5-aza injection, the regeneration was progressing. It is noted that no significant difference in ABR threshold shifts was observed between the 5- and 6-week groups, which may indicate that the hair cell functionality regeneration reaches stability from 5 weeks after 5-aza injection.

DPOAE thresholds of the 5-aza mice at 16 and 24 kHz progressively improved from 5 to 16 dB from 1 to 6 weeks following surgery (Figure 4C). Furthermore, previous data revealed that the percentage of mice with regenerated hair cells increased from 1 to 2 weeks after the treatment and then decreased from 4 to 6 weeks following 5-aza treatment (Deng et al., 2019). Here, DPOAE thresholds in the 5-aza-treated mice exhibited continuous improvement from 1- to 6-week postsurgery in both frequencies. The reason for the inconsistency is obscure, but it may be because not all the regenerated outer hair cells are functional and that the functionality of new hair cells may not be linear with the number of new hair cells. Further experiments are required to understand the relationship between regenerated hair cells and their functionality. There are greater DPOAE threshold improvements at 24 kHz than 16 kHz by 6 weeks after 5-aza treatment (Table 3), which may indicate a better functionality of outer hair cells located in a relatively higher frequency area. Statistical analysis reveals that the 5-aza group showed significantly improved thresholds compared to the vehicle groups from 1 to 6 weeks after treatment in both frequencies. Within the 5-aza group, the threshold improvement of the 5- and 6-week groups was greater than the 1-week group (Table 4). These data may also suggest a continuously improved functionality of outer hair cells following 5-aza treatment.

In our previous study, the relative Dnmt1 mRNA expression levels were measured by quantitative real-time PCR in the 5-aza and vehicle treatment groups 2 weeks after the inner ear injection. The results showed that the Dnmt1 mRNA expression level of the 5-aza group was significantly lower than that of the vehicle group, suggesting that DNA demethylation and Dnmt1 inhibition may play a role in 5-aza-induced new hair cell generation (Deng et al., 2019). In this study, hearing function improvements were observed in the 5-aza group, but not in the vehicle treatment group. These data suggest that the hearing function improvement observed in this study may be related to 5-aza-induced DNA demethylation. Independent research is required to further detail the relationship between hearing function improvement and DNA demethylation.

This study aimed to determine the function of regenerated adult mammalian auditory hair cells stimulated by 5-aza, an epigenetic reprogramming approach. The results of this study suggest that the DNA demethylating agent 5-aza is capable of regenerating hair cells with recovered functionality, including expression of cell type-specific functional proteins, ribbon synapse proteins, and hearing functional improvement. This study may open avenues to develop novel methods to restore the function of mammalian hair cells to treat hearing loss. Outcomes of this study may be applied to other neurodegenerative diseases, which may provide insights into translation research to generate functional cells *via* epigenetics-based reprogramming.

## Data Availability Statement

The original contributions presented in the study are included in the article/supplementary material, further inquiries can be directed to the corresponding author.

## Ethics Statement

The animal study was reviewed and approved by the Wayne State University Institutional Animal Care and Use Committee.

## Author Contributions

XD and ZH designed the project and wrote the manuscript, performed the experiment, and analyzed the data. Both authors have read the article and approved the final version of the manuscript.

## Conflict of Interest

The authors declare that the research was conducted in the absence of any commercial or financial relationships that could be construed as a potential conflict of interest.

## Publisher’s Note

All claims expressed in this article are solely those of the authors and do not necessarily represent those of their affiliated organizations, or those of the publisher, the editors and the reviewers. Any product that may be evaluated in this article, or claim that may be made by its manufacturer, is not guaranteed or endorsed by the publisher.
